# The emerging roles of SUMOylation in the tumor microenvironment and therapeutic implications

**DOI:** 10.1186/s40164-023-00420-3

**Published:** 2023-07-06

**Authors:** Yunru Gu, Yuan Fang, Xi Wu, Tingting Xu, Tong Hu, Yangyue Xu, Pei Ma, Qiang Wang, Yongqian Shu

**Affiliations:** 1 Department of Oncology, The First Affiliated Hospital of Nanjing Medical University, 300 Guangzhou Road, 210029 Nanjing, People’s Republic of China; 2 Jiangsu Key Lab of Cancer Biomarkers, Prevention and Treatment, Collaborative Innovation Center for Cancer Personalized Medicine, Nanjing Medical University, Nanjing, China; 3 Department of Hepatobiliary Surgery, The First Affiliated Hospital of Anhui Medical University, 230022 Hefei, Anhui Province People’s Republic of China

**Keywords:** Tumor microenvironment, Post-translational modification, SUMOylation, Hypoxia, Metabolism, Inflammation, Immune response, Clinical implications

## Abstract

Tumor initiation, progression, and response to therapies depend to a great extent on interactions between malignant cells and the tumor microenvironment (TME), which denotes the cancerous/non-cancerous cells, cytokines, chemokines, and various other factors around tumors. Cancer cells as well as stroma cells can not only obtain adaption to the TME but also sculpt their microenvironment through a series of signaling pathways. The post-translational modification (PTM) of eukaryotic cells by small ubiquitin-related modifier (SUMO) proteins is now recognized as a key flexible pathway. Proteins involved in tumorigenesis guiding several biological processes including chromatin organization, DNA repair, transcription, protein trafficking, and signal conduction rely on SUMOylation. The purpose of this review is to explore the role that SUMOylation plays in the TME formation and reprogramming, emphasize the importance of targeting SUMOylation to intervene in the TME and discuss the potential of SUMOylation inhibitors (SUMOi) in ameliorating tumor prognosis.

## Background

Over the past decade, the variety of protein PTMs has rapidly increased. PTMs alter targeted proteins’ conformation, charge state, and hydrophobicity, affecting their stability and activity. Diverse genomic and proteomic alterations are required in the process that a normal cell converts to a malignant one. Several dysregulated PTM pathways can result in cancer cell proliferation and metastasis. Nowadays, hundreds of unique PTMs in eukaryotic cells have sprung up, such as acetylation, phosphorylation, ubiquitination, and SUMOylation.

PTM with SUMO, named SUMOylation, was discovered in the middle of the 1990s [1]. Observed in numerous human pathologies, particularly in tumorigenesis and progression, SUMOylation acts in various aspects of cell biological process, such as DNA damage repair, protein trafficking, and cell cycle regulation [2, 3, 4, 5].

In our former research, we discussed the connection between epigenetic modifications and TME. For instance, distinct features of the TME tend to alter the activity of enzymes engaged in RNA methylation while each form of methylation contributes to the formation of the different TME states, including hypoxia, metabolic dysregulation, chronic inflammation, and immune escape [6]. Notably, recent studies have found that many RNA methylation-related enzymes could be SUMOylated, leading to the promotion or impression of tumor progression. The N6-Methyladenosine (m^6^A) methyltransferase activity of methyltransferase 3 (METTL3) is inhibited due to its SUMOylation by SUMO1 at residue K211, K177, K212, and K215, resulting in the decrease in m^6^A levels and increase in mRNA degradation that promotes tumorigenesis [7]. Similarly, it has been confirmed that a “reader protein” of m^6^A methylation named YTHDF2 (YTH N6-methyladenosine RNA binding protein 2) was SUMOylated at residue K571 according to microenvironmental hypoxia. SUMOylation of YTHDF2 increases the binding affinity to m^6^A sites and promotes the degradation of several tumor-related mRNAs [8]. Not only the enzymes of m^6^A, but also 5-methylcytosine (m^5^C) methylation such as NOP2/Sun RNA methyltransferase 2 (NSUN2) can be SUMOylated. The carcinogenic activity of NSUN2 is promoted by SUMOylation, mediating its stability and nuclear transport [9].

It is well known that the TME mediates interactions among proteins due to subtle changes such as oxygen content, PH, and ATP, which can potentially expose peptide motifs that are the main factor affecting interactions between SUMOylation ligase and targeted proteins, including transcription factors (TFs) and oncoproteins. To date, studies on SUMOylation have concentrated on carrying out proteomics and identifying targeted proteins with specific lysine (K) residues and motifs, but not the SUMOylation stoichiometry in the TME, which differed from each type of tumor [10]. Here, we display the crucial role of SUMOylation in various cancers and deliberate on the SUMOylation activity in distinct TME surrounding the enzyme-protein substrate and the potential ability of SUMOylation to affect the TME.

### Chemical basis of SUMOylation

**Fig. 1:**
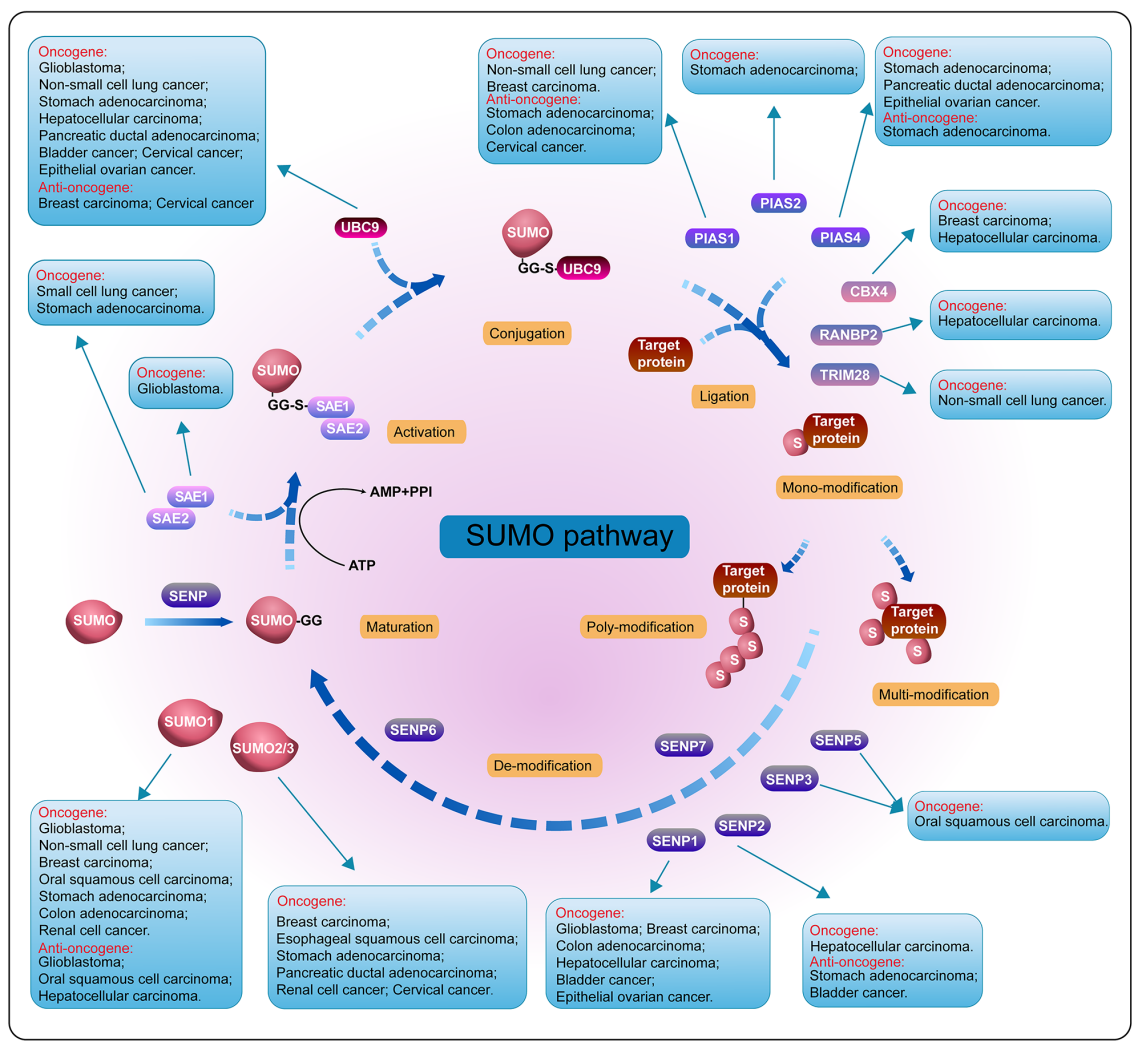
The SUMO procedure diagram and its function in various tumors. SUMO proteins are firstly processed by SENP, and after the formation of a high-energy thioester bond between the C-terminal SUMO and active site cysteine of SAE1/2, activated SUMO is then transferred to a cysteine residue in the active site of Ubc9. It is finally transferred to a target lysine with the help of Ubc9 and SUMO E3 ligase. The expression and functions in different tumors of SUMO E1, E2, E3 ligases and SENPs are also depicted in this figure

The SUMOylation process is similar to ubiquitination: it includes maturation, activation, conjugation, and deconjugation steps. (Fig. 1). In mammals, SUMO proteins begin as inactive precursors, which are firstly processed by sentrin-specific proteases (SENPs). The C-terminal di-glycine (-GG) motif of SUMO is subsequently exposed by SENPs, thus activating SUMOs. Heterodimeric E1 enzyme (SAE1/2) forms a high-energy thioester bond between its cysteine site and the C-terminal of SUMO in an ATP-dependent manner. Secondly, activated SUMOs are transferred to a cysteine residue of SUMO conjugating enzyme (UBC9, a SUMO E2 ligase). Finally, with the help of UBC9 and SUMO E3 ligase, they are conjugated to a target lysine that is usually in a consensus sequence (ψ-K-X-D/E, ψ: large hydrophobic amino acid; K: lysine; X: any amino acid; D: aspartate; E: glutamate). SUMO E3 ligases are another class of proteins in the SUMOylation cascade, mainly including members of the protein inhibitors of activated signal transducer and activator of transcription (PIAS) family [11, 12]. Although the SUMO enzymes are fewer in number than their counterparts in the ubiquitination pathway, the amount of SUMO substrates are strikingly large, affecting cells activity and disease development. Furthermore, SUMO maturation and deconjugation are both carried out by SENPs. SENP family comprises six members in mammals (SENPs 1–3 and 5–7), and SENPs 1–3 and 5 are the major executors while SENP 6–7 remove the SUMO monomers from the polymeric SUMO chains. Apart from covalent SUMO modification, non-covalent interaction between SUMOs and target protein happens thanks to the SUMO-interacting motifs (SIMs), which contain a hydrophobic core binding with the surface region of SUMO [13].

## Multiple functions of SUMOylation in cancers

### Neural system tumors

The SUMOylation cascade modifies targeted proteins reversibly and dynamically, and participants of SUMO circulation play distinct roles in various tumors (Fig. 1; Table 1). In glioblastoma (GBM), proteins involved in the SUMOylation cascade are upregulated, such as E1 (SAE1), E2 (UBC9) components, and SENP1, promoting tumor progression [14, 15]. Researchers found that CRMP2 SUMOylation induced by UBC9 could significantly promote GBM cell proliferation [16]. SUMOylation of promyelocytic leukemia (PML) protein promoted by prolyl-isomerase Pin1 facilitates c-Myc proteins stability, promoting glioma stem cells (GSCs) maintenance and GBM malignancy [17]. SUMO1 conjugation of FUS boosted by lncRNA RMST enhances the interaction between FUS and heterogeneous nuclear ribonucleoprotein D (hnRNPD), maintaining their expression and inhibiting tumorigenesis [18].

**Table 1:** Molecular mechanism and biological functions of SUMO enzymes in diverse cancers

Cancer	SUMO enzyme(s)	Substrate	Biological mechanisms and functions	Refs
GBM	UBC9	CRMP2	Promote cancer cell proliferation	[16]
	SUMO1	PML	Promote tumor malignancy via stabilizing c-Myc and GSCs maintenance	[17]
	SUMO1	FUS	Inhibit tumorigenesis via alleviating cells mitophagy	[18]
	SAE1,UBC9,SENP1	NR	Promote cancer cells proliferation and migration and inhibit apoptosis	[15]
NSCLC	SUMO1	YTHDF2	Promote tumor progression via increasing its binding affinity with m^6^A -modified mRNAs	[19]
	SUMO1	METTL3	Promote tumor progression via decreasing its m^6^A methyltransferase activity	[7]
	TRIM28	IRF	Promote tumorigenesis via regulating tumor microenvironment	[20]
	UBC9,PIAS4	SIRT1	Promote tumor progression via facilitating EMT	[21]
	UBC9,PIAS4,SUMO1	SLUG	Promote tumor progression via enhancing HDAC1 recruitment and metastasis	[22]
	PIAS1	PML	Promote tumorigenesis via increasing proteasome-mediated degradation of PML	[23]
	SUMO1	VEGFR2	Inhibit tumor progression via suppressing angiogenesis, proliferation, and migration.	[24]
SCLC	SAE2	NR	Promote tumor progression only in high c-Myc expression group	[25]
BRCA	UBC9	NR	Inhibit tumor malignancy through being upregulated by FOXP3	[26]
	SUMO2/3	MYC	Promote cancer cell metastasis	[27]
	SENP3	AKT1	Promote macrophage polarization via AKT1 phosphorylation and activation	[30]
	SENP1	GATA1	Promote metastasis and invasion via facilitating CSN5-mediated ZEB1 degradation	[31]
	SENP1	MYC	Promote tumorigenesis via decreased ubiquitination and stabilization of c-MYC	[29]
	CBX4,SUMO1	HTERT	Promote metastasis through facilitating EMT and repressing E-cadherin expression	[32]
	PIAS1	SNON	Promote metastasis via increasing EMT	[33]
OSCC	SUMO1	SMAD4	promote tumor progression via TGFβ1-induced SUMO1 conjunction	[34]
	SUMO1	MDM2	Promote tumor aggressiveness through increasingmdm2 expression	[35]
	SUMO1	PTEN	Inhibit tumor progression via suppressing AKT/mTOR signaling pathway	[36]
	SUMO1	SP1	Inhibit tumor progression and promote radiosensitization through decreasing SP1 activity and promoting PTEN transcription in a BetA-dependent manner	[37]
	SENP3,SENP5	NR	Promote tumorigenesis via regulating differentiation of cancer cells	[38]
ESCC	SUMO2/3	MCM10	Promote cancer cell proliferation and metastasis	[40]
	SUMO2/3	HSP27	Promote proliferation and migration via PKM2 upregulation and E-cadherin downregulation	[41]
STAD	PIAS2	P38	Promote metastasis via forming a positive loop feedback with ROS accumulation	[42]
	PIAS1,PIAS4	NR	Decrease risk of mortality among patient receiving second-line docetaxel-based therapy	[43]
	SUMO2/3	NSUN2	Promote tumor progression via maintaining its stability and methyltransferase activity	[44]
	SENP2	NDRG2	Inhibit tumor progression via stabilizing NDRG2	[45]
	UBC9,SUMO1	IGF-1R	Promote proliferation and migration via increasing transcription activity of SNAI2	[46]
	SAE2	NR	Promote tumor aggressiveness	[47]
COAD	SENP1	p21,p27	Promote cell growth through downregulating CDK inhibitors and suppressing G1 arrest	[48]
	SUMO1	p53	Promote cancer cell metastasis	[49]
	PIAS1	IRF-1	Inhibit tumor progression via IRF-1 nuclear translocation and subsequent apoptosis	[50]
HCC	UBC9,SUMO1	PKM2	Promote tumor progression via PKM2 excretion and TME reprogramming	[51]
	RANBP2	FTO	Promote cancer malignancy via downregulating GNAO1 mRNA expression	[52]
	SENP2	HNRNPK	Promote tumorigenicity via disrupting interaction between HNRNPK and p53	[53]
	SUMO1	MANF	Inhibit tumor progression through inhibiting the NF-κBNF-κB/Snail signal pathway	[54]
	UBC9,SUMO1	METTL3	Promote tumor metastasis via facilitating Snail expression in an m6A-dependent manner	[55]
	SUMO1	NRF2	Promote tumor malignancy via promoting de novo serine synthesis	[56]
	CBX4	HIF1α	Promote angiogenesis via enhancing HIF-1α transcriptional activity and VEGF expression	[57]
	SENP1	HIF1α	Promote tumorigenesis through enhancing hypoxia-induced stemness	[58]
PDAC	PIAS4	VHL	Promote tumor progression via inhibiting VHL-mediated HIF1α degradation	[60]
	SUMO2,UBC9	RNF40	Promote tumor aggressiveness via resolving DNA double-strand breaks	[61]
BLCA	SENP1	NR	Promote tumor recurrence	[62]
	SENP2	TBL(R)1	Inhibit metastasis via suppressing β-catenin nulear translocation and MMP13 activation	[63]
	UBC9	HNRNPA1	Promote lymphangiogenesis and LN metastasis via facilitating SOX18 transcription	[64]
RCC	SUMO1	HAF	Promote tumor progression through inducing HIF-2 transactivation	[65]
	SUMO2	VHL	Promote angiogenesis via HIF-α degradation inhibition	[66]
PC	UBC9	STAT4	Promote PCA progression via enhancing immunosuppressive phenotype of TAMs	[67]
CCA	UBC9,SUMO2/3	HADA3	Promote tumorigenesis through HPV 16E6 stimulation	[68]
	UBC9,PIAS1	FOXM1B	Inhibit HPV carcinogenesis through disturbing cell cycle process	[70]
EOC	UBC9	NR	Promote cancer cell proliferation via facilitating PI3K/AKT signaling pathway	[71]
	SENP1	HIF1α	Promote tumor progression via weakening sensitivity to chemotherapy	[72]
	PIAS4	SP1	Promote metastasis via suppressing SIRT1 transcription and enhancing EMT	[72]

GBM: glioblastoma; NSCLC: non-small cell lung cancer; SCLC: small cell lung cancer; BRCA: breast carcinoma; OSCC: oral squamous cell carcinoma; ESCC: esophageal squamous cell carcinoma; STAD: stomach adenocarcinoma; COAD: colon adenocarcinoma; HCC: hepatocellular carcinoma; PDAC: pancreatic ductal adenocarcinoma; BLCA: bladder cancer; RCC: renal cell cancer; PC: prostate cancer; CCA: cervical cancer; EOC: epithelial ovarian cancer

### Respiratory tumors

The binding affinity of YTHDF2 with m^6^A motif can be significantly increased by SUMOylation while SUMO1-modulated METTL3 SUMOylation significantly suppresses its m^6^A methyltransferase activity, resulting in lung cancer progression [7, 19]. TRIM28, an uncommon protein in SUMOylation cascade, is overexpressed in lung adenocarcinoma with low immune and stromal scores, regulating the TME by SUMOlating the IRF family proteins [20]. UBC9/PIASy-mediated SUMOylation decreases sirtuin 1 (SIRT1) and increases transcriptional repression activity of SLUG, predicting more invasive types of lung cancers [21, 22]. PIAS1-induced SUMOylation of PML facilitates its degradation and thus promotes NSCLC progression, while vascular endothelial growth factor receptor 2 (VEGFR2) SUMOylation inhibits angiogenesis signaling pathway in NSCLC [23, 24]. Additionally, SAE2 was found highly expressed in small cell lung cancer (SCLC), promoting migration and invasion, and decreasing sensitivity to chemotherapy [25].

### Mammary system tumors

Researchers showed that UBC9 was upregulated through Forkhead Box Protein P3 (FOXP3), a tumor-suppressing TF, which could act as a novel activator of SUMOylation in Breast Cancer (BRCA) [26]. When compared with nonmetastatic cells, metastatic breast cancer cells exhibit an upregulated SUMO2/3 modification profile, especially on MYC. SAE1/2 enzymatic activity losing facilitates MYC synthetic lethality while SENP1-driven deSUMOylation decreases its ubiquitination-mediated degradation [27, 28, 29]. SENP3-deficiency-mediated AKT1 SUMOylation leads to AKT1 hyper-phosphorylation and activation, promoting macrophages’ M2 polarization [30]. M2-type macrophages interact with tumor cells by releasing EGF, MMPs, VEGF, and TGFβ, thus promoting tumor proliferation, invasion, angiogenesis, and immune escape. In triple-negative breast cancer (TNBC), overexpressed SENP1 promotes CSN5-mediated ZEB1 protein degradation via deSUMOylation of GATA1, and ultimately enhances metastasis and invasion of tumor cells [31]. The catalytic component of the human telomerase enzyme (HTERT) was reported to be SUMOylated by SUMO1 and CBX4, and PIAS1 and TIF1γ cooperated to facilitate the SUMOylation of SnoN, both triggering the EMT program [32, 33].

### Digestive system tumors

SUMO1 conjugation to SMAD4 and murine double minute 2 homolog (MDM2) increase their expression and are involved in oral squamous cell carcinoma (OSCC) aggressiveness [34, 35]. Through its PDZ domain, Regulator of G protein signaling 12 (RGS12) upregulates SUMOylation of phosphatase and tension homolog (PTEN). Moreover, specificity protein 1 (SP1) SUMOylation can activate PTEN transcription to block the AKT/mTOR signaling pathway, increasing radio sensitization of OSCC cells [36, 37]. SENP3 and SENP5 were found overexpressed and related to differentiation of OSCC [38, 39]. A germline variant of minichromosome maintenance proteins (MCMs) can increase its SUMOylation levels, facilitating Esophageal squamous cell carcinoma (ESCC) proliferation and metastasis [40]. Modified by SUMO2/3, heat shock protein 27 (HSP27) is upregulated, which increases pyruvate kinase isoenzyme M2 (PKM2) and decreases E-cadherin, enhancing the malignant extent of ESCC cells [41].

SUMOylation was found to create a positive feedback loop in gastric cancer (GC). Wang et al. found that as a result of P38α-SUMOylation, reactive oxygen species (ROS) accumulated, which facilitated p38α-SUMOylation by improving the PIASxα protein stability, creating a favorable environment for survival and metastasis [42]. PIAS1 and PIAS4 show a higher level among patients with a lower risk of mortality after second-line docetaxel-based chemotherapy [43]. SUMO-2/3 modifies NSUN2 and modulates its stability and nuclear transport, promoting its carcinogenic activity [44]. Additionally, SENP2 was suggested to stabilize N-myc downstream-regulated gene 2 (NDRG2) which functioned as a tumor suppressor gene [45]. UBC9/SUMO1-mediated insulin-like growth factor 1 receptor (IGF-1R) SUMOylation increases transcription activity of its substrate proteins, including snail family zinc finger 2 (SNAI2) [46]. SAE2 was also substantiated to play a crucial role in the aggressiveness of GC and predict a poor survival outcome [47].

SENP1 is overexpressed in colon cancer (COAD) and silencing of SENP1 inhibits cell proliferation through upregulating CDK inhibitors including p21 and p27 [48]. Besides, 46 cases of colon cancer were investigated, showing that patients who exhibited high expression of SUMO1 and SUMOylated P53 were more likely to experience metastasis [49]. Contrary to many other cancers, PIAS1, which was found associated with IRF-1 nuclear translocation and apoptosis, was downregulated in colon cancers [50].

In hepatocellular carcinoma (HCC) cells, SUMOylation reprograms the TME to affect cancer-TME crosstalk. Tumor-infiltrating macrophages, one of the most abundant stromal cell types in the HCC TME, inhibit anti-tumor immunity by inducing matrix remodeling, angiogenesis, and tumor metastasis. UBC9/SUMO1-mediated PKM2 SUMOylation induces its exosomal excretion, which triggers monocyte-to-macrophage differentiation to substantially increase the abundance of macrophages to help tumor cell invasion and metastasis [51]. RANBP2, a SUMOylation E3 ligase, mediates Fat mass- and obesity-associated gene (FTO) SUMOylation and subsequent degradation, inducing Guanine nucleotide-binding protein G (o) subunit alpha (GNAO1) instability, an m^6^A substrate of FTO and a tumor suppressor in HCC [52]. P53-stabilizing and activating RNA (PSTAR), a newly found lncRNA, enhances SUMO1-dependent SUMOylation of heterogeneous nuclear ribonucleoprotein K (hnRNP K) and disrupts its deSUMOylation through SENP2, facilitating transactivation of p53 [53]. SUMO1 also promotes Mesencephalic astrocyte-derived neurotrophic factor (MANF) nuclear translocation and enhances its interaction with p65, inhibiting the nuclear factor kappa B (NF-κB)/Snail signal pathway in EMT [54]. UBC9/SUMO1-mediated METTL3 SUMOylation regulated Snail expression and HCC metastasis in an m^6^A-dependent manner [55]. Nuclear factor erythroid-2 related factor 2 (NRF2) was found to be SUMOylated by SUMO1 at K110, promoting de novo serine synthesis and HCC tumorigenesis [56]. It was suggested that CBX4 could enhance VEGF expression in HCC cells and promote angiogenesis through enhancing hypoxia-inducible factors (HIF-1α) SUMOylation and its transcriptional activity [57]. Intriguingly, SENP1 augments the transcriptional activity of HIF-1α under hypoxic depending on deSUMOylation function. Meanwhile, a positive feedback loop was demonstrated between HIF-1α and SENP1, resulting in stemness and tumorigenesis of HCC [58].

Researchers detected coexpression of MYC and SUMO-related factors (such as SAE1, UBC9, SUMO1, SUMO2/3) in Pancreatic ductal adenocarcinoma (PDAC), and hyperactivation of MYC was associated with increased sensitivity to pharmacological SUMO inhibition, which provided a new therapeutic strategy to PDAC [59]. PIAS4 contributes to von Hippel-Lindau (VHL) SUMOylation and impairs its function, upregulating HIF1α and its targets including VEGF and STAT3 [60]. UBC9/SUMO2-regulated SUMOylation of DNA double-strand breaks (DSB) repair proteins (e.g. RNF40) sustains their stability, maintaining the DNA damage response (DDR) [61].

### Urinary system tumors

SENP1 is upregulated in urinary content and is regarded as a predictor of the recurrence of bladder cancer (BLCA) [62]. While SENP2 is downregulated in BLCA. Mechanistically, SENP2 inhibits nuclear translocation of β-catenin through deSUMOylation of TBL1/TBLR1, inhibiting the MMP13 activation and BC cell metastasis [63]. UBC9 overexpression induced by lncRNA ELNAT1 catalyzes SUMOylation of hnRNPA1, promoting ELNAT1 packaged into EVs and activating SOX18 transcription to induce lymphangiogenesis [64].

In renal cell cancer (RCC), hypoxia-associated factor (HAF)-mediated HIF-2 transactivation requires SUMOylation of HAF by SUMO1 in an oxygen-sensing manner, contributing to the maximal induction of HIF-2 target genes and tumor progression [65]. RWD domain-containing protein SUMO Enhancer (RSUME) was found to SUMOylated VHL to alleviate HIF degradation, favoring RCC proliferation. Additionally, the capability of binding between HIF and VHL of RSUME was found dependent on VHL SUMOylation, specifically SUMO2 [66].

In prostate cancer (PC), UBC9 mediates SUMOylation of signal transducer and activator of transcription 4 (STAT4) on K350 which activates the immunosuppressive phenotype of tumor-associated macrophages (TAMs), while macrophage-specific UBC9 ablation can facilitate STAT4-induced interplay among TAM-CD8(+) T cells-cancer cells to curb tumor progression [67].

### Female reproductive system tumors

In cervical cancer (CCA), Human Papilloma Virus (HPV) 16E6 targets hADA3 for SUMOylation-mediated degradation [68, 69]. Interaction between Forkhead box M1b (FOXM1b) and UBC9/PIAS1 can be impaired by HPV16 E7, decreasing SUMOylation of FOXM1b and sustaining its stability [70].

Dong et al. have demonstrated that UBC9 overexpression could significantly increase the proliferation of Epithelial Ovarian Cancer (EOC) through PI3K/AKT signaling pathway [71]. SENP1 was shown to upregulate HIF-1α expression through deSUMOylation, increasing cisplatin resistance in EOC cells [72]. Furthermore, PIAS4 was confirmed to induce SUMOylation of SP1, a transcriptional activator, and prevent it from binding to the SIRT1 promoter, downregulating it and impeding EMT [72].

### SUMOylation takes part in the hypoxic response pathway

A hypoxic TME is a pivotal hallmark of most solid tumors. Under hypoxia, dramatic reprogramming of biological processes happens, including anaerobic energy production, lipid metabolism modulation, oxygen delivery increase, etc. Those dysregulation gene expressions in distinct events also contribute to tumor cell ethology alteration through the activation of HIFs [73, 74]. Fine-tuning of the SUMO conjugation machinery extensively occurs under various stress conditions, especially hypoxia, regarded as a homeostatic mechanism evolving in multicellular organisms to sustain cellular and tissue functions [75, 76, 77]. SUMO pathway in cancer cells is also dysregulated under the catalysis of hypoxia, facilitating proliferation, invasion, metastasis, and even resistance to chemotherapy as mentioned above.

HIF-mediated target gene activation was depicted in researches. Under normoxia, HIF is modified by pVHL, an E3 ubiquitin ligase, through hydroxylation activity of the prolyl hydroxylase domain containing proteins (PHDs) and factor inhibiting HIF (FIH), leading to subsequent proteasomal degradation. In the hypoxic TME, the decreased hydroxylation facilitates HIFα nuclear transport, dimerization with HIFβ, and recruitment of CBP/p300 coactivators. As a result, hypoxia-targeted genes are stimulated via HIF binding to hypoxia responsive elements (HRE) [78]. Effect of PTM during cellular response to hypoxia has been extensively studied and SUMO hemostasis was indispensable to plenary activation of hypoxia signaling. A significant increase in SUMO1 mRNAs and proteins was observed, and interaction between SUMO1 and HIF1α was also demonstrated under hypoxic conditions such as pulmonary hypertension [79, 80].

However, a SILAC-quantitative proteomic-based study suggested that it was not SUMO1, or SUMO2/3 increase but a massive augment in SUMOylation status that altered under hypoxia. Identically, based on comparative mass spectrometry, it was illustrated that oxygen concentration altered the activity of enzymes in the SUMO pathway, especially the SENP family (SENP1 and SENP3). Intriguingly, a few SUMO enzymes also act as hypoxia-induced SUMO1 targets that change under the TME, including RanBP2 and PIAS2 [81, 82].

**Fig. 2:**
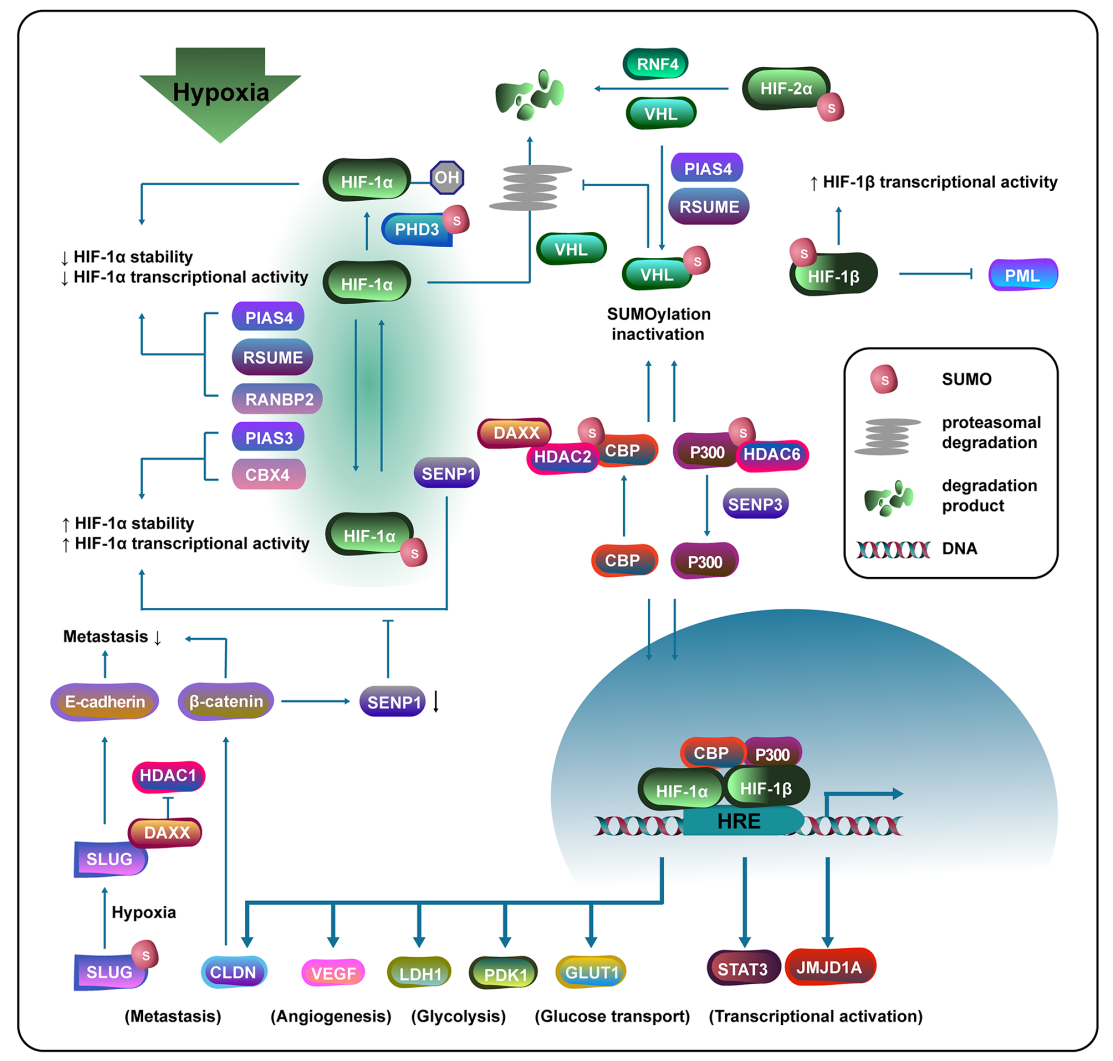
Crosstalk between SUMOylation related enzymes and hypoxia signaling pathway. SUMOylation related enzymes regulate HIF-1a stability and transcriptional activity through directly mediating its SUMOylation and indirectly influencing other participants involved in hypoxia signaling pathway, causing expression level alteration of critical genes that modulating cancer cells biological processes such as metastasis, angiogenesis, and glycolysis. Meanwhile, hypoxia can also affect expression of some actors via monitoring their SUMOylation state

Researchers have found that HIF, the most significant factor in the hypoxic response pathway, was widely modified by SUMOylation, influencing its stability and transcriptional activity (Fig. 2). HIF-1α is upregulated through SUMO1 modification at K391/K477, and overexpression of PIAS3 can maintain its stability and transcription activity [83, 84, 85]. In agreement, Li et al. discovered that E3 ligase activity of CBX4 SUMOylated HIF-1α, promoting its transcriptional activity and expression of VEGF, which facilitated tumor malignancy via angiogenesis [57, 86]. While in other studies, SUMO E3 ligase (RanBP2 and PIAS4) were found negatively regulate hypoxia-mediated HIF-1α stability and transactivation [87, 88]. In some conditions, SENP1-mediated deSUMOylation also increases HIF-1α transcriptional activity and facilitates HIF1α-dependent genes expressions such as VEGF and glucose transporter 1 (GLUT1) [89]. The SENP1/HIF-α positive feedback loop illustrated by Cui et al. mentioned above also support this scenario [58]. The reason for the controversial scenario might be the fact that several factors among the hypoxia pathway were regulated via the alteration of SUMO enzymes, resulting in distinct HIF-1α activity. Additionally, HIF-2α was found modified by SUMOylation at K394, leading to VHL and RNF4 (SUMO-targeted ubiquitin ligases)-mediated proteasomal degradation [90]. Tojo et al. elucidated that the aryl hydrocarbon receptor nuclear transporter (ARNT), also named HIF-1β, was modified by SUMO1 at K245, disturbing interaction with PML and augmenting the transcriptional activity of ARNT [91].

Meanwhile, several participants apart from HIF in hypoxia signaling were suggested to be regulated by SUMOylation (Fig. 2). HIF1α E3 ligase HAF degrades HIF-1α in a SUMOylation manner, while hypoxia-induced SUMOylation of HAF enables its HIF-2α combination to enhance transcriptional activity [65]. Induced by hypoxia, RSUME enhances overall SUMO1-3 conjugation, thus promoting the stabilization of HIF-1. In addition, RSUME SUMOylates and physically interacts with pVHL, thus suppressing the complex aggregation of pVHL, Elongins and Cullins (ECV), subsequently alleviating ubiquitination-induced degradation of HIF-1/2α [92, 93]. PIAS4-induced VHL SUMOylation by SUMO1 on K171 facilitates VHL oligomerization and abrogates its inhibitory function on HIF-1α activity. Therefore, target genes of HIF-1α such as JMJD1A, VEGF, and STAT3 are activated, and tumor progression is promoted [60, 94] .PHD3 SUMOylation by SUMO2/3 was also found to repress HIF-1-dependent transcriptional activity [95]. Furthermore, p300 and CREB binding protein (CBP) act as homologous transcriptional coactivators. K1017 and K1029 of p300 are modified by SUMO1, leading to the recruitment of histone deacetylase 6 (HDAC6), and SUMOylation deficiency via SENP3 increases p300-mediated transcriptional activity [96, 97]. Hypoxia/SIRT1-mediated UBC9-K65 acetylation reduction promotes CBP SUMOylation as well as hypoxia signaling cascade [98]. On the contrary, Kuo et al. exhibited that CBP could be SUMOylated by SUMO1 at K999, K1034, and K1057, negatively regulating its translational activity through interacting with death domain-associated protein (Daxx), which recruited HDAC2 and played a role of transcriptional corepressor [99].

Moreover, the invasive ability of cancer cells is likely to be regulated by SUMOylation. Hypoxia increases Slug SUMOylation by the way of disrupting its crosstalk with SENP1 and SENP2, promoting lung cancer metastasis. Also, Xie et al. showed that Slug SUMOylation could be stabilized by p14 (ARF), promoting EMT [22, 100]. SUMOylation-dependent negative feedback between HIF-1α and CLDN6 has been elucidated by researchers. CLDN6, transcriptionally upregulated by HIF-1α, was suggested to combine β-catenin, leading to β-catenin degradation and preventing its nuclear translocation. β-catenin acts as a TF of SENP1, and degradation of it downregulates SENP1, causing HIF-1α SUMOylation and degradation [101].

### SUMOylation influences metabolism signaling pathways

Metabolic stress, in addition to other stress, results in changes in endogenous synthesis and exogenous uptake of nutrients, which are fuels for various biological procedures, including protein modification. Crosstalk between SUMOylation and metabolic dysregulation can partly explain how malignant cells thrive in the TME. SUMOylation has been extensively studied in metabolic diseases, especially diabetes. SENP1 was suggested to amplify insulin exocytosis, NADPH generation, and subsequent glutathione (GSH) reduction, rescuing β cell function in type 2 diabetes [102]. In support, SENP1 can ameliorate type 1 diabetes. Mechanistically, SENP1 facilitates deSUMOylation of NEMO, in adipocytes, to alleviate NF-κB activity and subsequent inflammation [103]. However, few studies have focused on SUMOylation in metabolic reprogramming, which was indispensable for TME formation.

**Fig. 3:**
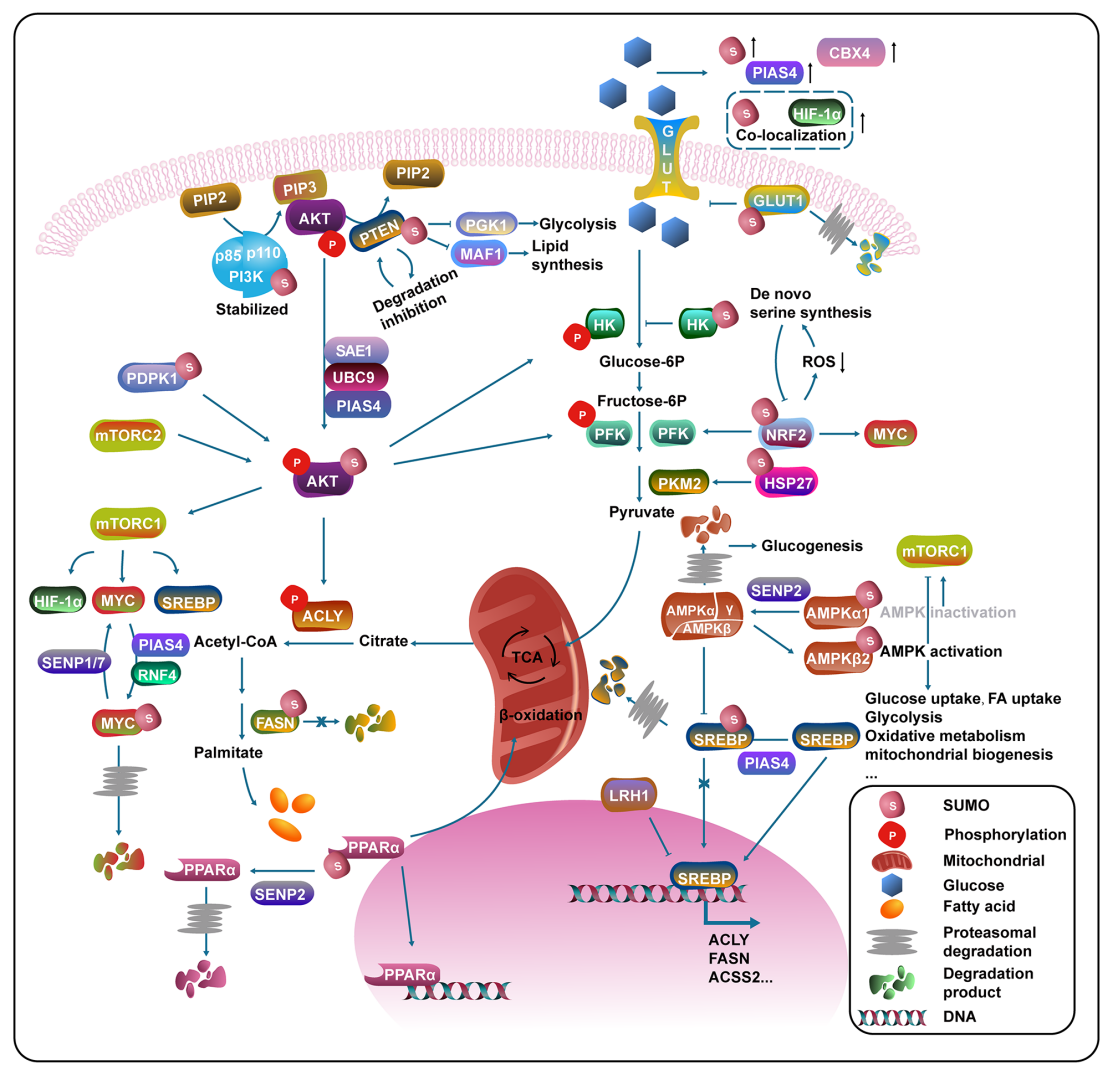
The role of SUMOylation in cancer metabolism pathways. This diagram focuses on SUMO modified metabolic enzymes, closely associated signaling pathways and TFs. Noteworthy, glucose metabolism is the most important metabolic pathway regulated by SUMOylation, contributing to metabolic reprogramming such as “Warburg effect”

According to the Warburg effect, despite oxygen-rich conditions, tumor cells tend to uptake glucose more rapidly and convert it to lactate via glycolysis because aerobic glycolysis helps malignant cells to survive. Features of the TME, such as hypoxia, hypoglycemic, and acidic microenvironment are closely related to functional proteins, whose SUMO modifications are nonnegligible [104]. SUMO modification alters tumor cell metabolism either directly via metabolic enzymes or indirectly via TFs and crucial signaling pathways (Fig. 3).

SUMOylation occurs on metabolic enzymes and dramatically alters the direction of metabolic flux (Fig. 3). Under hypoxic stress, SUMOylation plays a role in facilitating glycolytic pathway reprogram in that SUMO1 overexpression shows a correlation with clustering of glycolytic enzymes. In turn, the high glucose microenvironment demonstrates not only augmented SUMO pathway factors, including SUMO1-4 and SUMO ligase E3 (CBX4 and PIAS4) levels but also enhanced colocalization of HIF-1α and SUMO [19, 83]. Mo et al. suggested that SUMO2 modification promoted GLUT1 degradation, ultimately inhibiting glycolysis [105]. SUMO-defective hexokinase 2 (HK2) enters mitochondria to phosphorylate glucose to form glucose-6-phosphate, enhancing both glucose consumption and lactate production, while SUMOylated HK2 impedes its activity [106]. Long-chain saturated fatty acids starting from acetyl-CoA and malonyl-CoA are catalyzed by fatty acid synthetase (FASN), and its SUMOylation inhibits its proteasomal degradation, altering lipid metabolism and eliciting oncogene activity [107]. HSP27 SUMOylation by SUMO2/3 enhances PKM2 expression, promoting glycolysis and tumor progression [41]. In addition, K110 SUMOylation of NRF2 increases de novo serine synthesis by enhancing ROS clearance and phosphoglycerate dehydrogenase (PHGDH) upregulation. Accordingly, serine deficiency can in turn promote NRF2 SUMOylation. Moreover, NRF2 promotes glycolysis through activating PFK and MYC [56, 108].

Metabolism-associated TFs are widely SUMOylated, including sterol regulatory element-binding proteins (SREBP), peroxisome proliferator-activated receptor alpha (PPARα), and HIF-1 (Fig. 3). PTM of SREBP regulates its expression and related de novo lipogenesis procedure, a significant metabolic process in tumor cells. SUMOylation of SREBP suppresses its transcriptional activity and promotes HDAC3 recruitment, inhibiting lipid uptake and synthesis [109]. Lee et al. found that PKA-mediated SREBP1 phosphorylation facilitated PIAS4-induced SREBP1 SUMOylation, leading to degradation of SREBP1 via ubiquitination [110]. Nuclear receptor superfamily (FXR, LXR, LRH-1, and PPAR) undergo SUMOylation as well [109, 111, 112, 113]. LRH-1, one of those nuclear receptors, is SUMOylated at K289, decreasing oxysterol binding protein-like 3 (OSBPL3) expression and subsequent SREBP-1 processing [114, 115]. Another SUMOylation-mediated TF, named PPARα, is meanwhile a metabolic mediator. SENP2-induced PPARα deSUMOylation displays upregulated degradation, alleviating fatty acid oxidation and target genes transcription [116, 117]. It is well-known that HIF-1 is a crucial modulator of metabolic reprogramming and simultaneously a downstream molecule of SUMOylation. Specifically, it increases the transcription of GLUT1, lactate dehydrogenase 1 (LDH1), and pyruvate dehydrogenase kinase 1 (PDK1) (Fig. 2), turning pyruvate to acetyl-CoA and lactate for the mitochondrial tricarboxylic acid (TCA) cycle [118, 119, 120, 121].

In addition to acting on metabolic enzymes and metabolism-related TFs, various classical signaling pathways functioning in cancer metabolism undergo SUMOylation (Fig. 3). Growth factors or insulin stimulation of phosphoinositide 3-kinase (PI3K) induces glycolytic enzyme aldolase A (ALDOA) release, potentiating AKT-independent glycolytic flux. Activation of Serine-threonine kinase AKT is sufficient to stimulate tumor microenvironmental aerobic glycolysis and thus exerts its promoting effect on tumor growth and metabolism of individual cells. PI3K/AKT manages crucial phases in glycolysis through the activation of specific enzymes that mattered in cellular metabolic reprogramming, such as rapamycin (mTOR) complex 1 (mTORC1) and forkhead box O (FOXO) family [122, 123, 124, 125] p110β, which is a catalytic subunit of PI3K, can be modified by SUMO1 and SUMO2, increasing its activation of glucose metabolism in an AKT-dependent or independent manner [126]. Ong et al. displayed that SUMO E1 enzyme SAE1 was related to dysregulated cancer metabolism and became a potential target in HCC therapy. Additionally, SAE1 companied by E2 UBC9 and E3 ligase PIAS4 mediates AKT SUMOylation majorly at K276, regulating AKT activation and promoting tumor progression, which can be reversed by SENP1 [127, 128, 129]. SUMOylated AKT then promotes a series of metabolic cascade through whatever rapid or lagging courses. AKT can either directly phosphorylate ACLY or indirectly initiate ACLY via the SREBP family of TFs activation, facilitating de novo lipid synthesis. Likewise, SREBP also promotes fatty acid and sterol synthesis through many other related enzymes, such as FASN and Acetyl-coenzyme A synthetase (ACSS2) [130, 131]. AKT is activated at T308 through phosphoinositide-dependent protein kinase 1 (PDPK1) and S473 by mTORC2, leading to increased glucose uptake and glycolysis [122]. PDPK1 is also SUMOylated, and nonSUMOylated PDPK1 tethers LC3 to the endoplasmic reticulum to trigger macroautophagy/autophagy, which acts as a crucial pathway for cell metabolism [132]. mTORC1 activation was suggested to stimulate glycolysis and de novo lipid biosynthesis by influencing the transcription activity of HIF1-α and SREBP1/2 [133]. A sensor of energy status: 5’-AMP-activated protein kinase (AMPK), which comprises a catalytic α, a scaffolding β and a nucleotide binding regulator γ. Noteworthy, starvation-induced activation of AMPK results in the upregulation of catabolic metabolism and nutrient uptake, becoming a bridge that links metabolism and the SUMO pathway [134]. Dou et al. illustrated AMPKα as another pivotal substrate of SENP2, negatively regulating gluconeogenesis. SENP2-induced AMPKα deSUMOylation triggers its ubiquitination, subsequently promoting gluconeogenesis and blood glucose [135]. mTORC1 can be activated by AKT while inhibited via AMPKα, and PIAS4-induced AMPKα1 SUMOylation suppresses AMPK activation towards mTORC1 signaling [135]. Conversely, modified by PIAS4 and SUMO2, AMPKβ2 activates the α2β2γ1 AMPK complex, which might be an antagonistic mechanism relating to the ubiquitination of AMPKβ2 [136]. The significant oncogene MYC is widely known as a TF as well as an increased downstream of PI3K-AKT signaling [137]. SUMOylation and deSUMOylation mediate MYC protein stabilization and activation through the activity of SUMO-related enzymes, including PIAS1, RNF4, SENP1, and SENP7 [29, 138, 139]. However, research illustrated that SUMOylation of MYC also mediated its oncogene activity and inhibition of SUMOylation might be a possible therapy for MYC-elicited cancers [28]. Furthermore, hypoxia-mediated SENP1 inactivation alleviates basic helix-loop-helix family member e40 (BHLHE40) deSUMOylation, leading to transcriptional repression of PGC-1α, a crucial metabolic regulator, contributing to metabolic strategies inhibition, such as mitochondrial biogenesis and oxidative metabolism [82]. PTEN, a pivotal tumor suppressor factor functioning to dephosphorylate PIP3 to regenerate PIP2 and resulting in subsequent PI3K-AKT signaling inhibition, exhibits SUMO modification at K266. SUMOylated PTEN shows a low ubiquitination level, and in turn, proteasome inhibition results in PTEN SUMOylation accumulation [140, 141]. PTEN illustrates indirect regulatory activity for glucose metabolism via PI3K/AKT pathway as well as the direct mediatory potential for glycolysis through phosphoglycerate kinase 1 (PGK1), which catalyzes one of the two ATP producing reactions in the glycolytic pathway [142]. Apart from glucose metabolism, PTEN can also repress intracellular lipid accumulation by coordinating with Maf1, a TF that restrains RNA synthesis and lipid biosynthesis [142].

Recent studies have found that noncoding RNAs participated in the SUMO pathway and were associated with metabolism in a new way. Mo et al. showed that circRNF13 bound to the 3’- Untranslated Region (3’-UTR) of the SUMO2 mRNA and stabilized it, facilitating GLUT1 SUMOylation and subsequent degradation, ultimately inhibiting glycolysis and nasopharyngeal carcinoma (NPC) progression [105]. SUMO pathway mediates the tumor metabolism and the TME in many ways while the research on the regulation of SUMOylation by metabolism changes is very limited, more studies are warranted to clarify how it acts on SUMO processes.

### SUMOylation involves in the inflammatory TME

There are many components of the TME, including the extracellular matrix and various structures, like vascularization and inflammatory infiltrates. Crosstalk between malignant cells and their surrounding microenvironment helps tumor cells to survive. Inflammatory or immune-related cytokines and cells play an indispensable role in regulating this interaction [143]. Critical peptide motifs functioning as PTM sites are exposed when subtle changes in the TME are triggered by infection-inflammation, such as ATP and PH [3]. Karhaosen et al. described that global SUMOylation (SUMO2/3 isoforms) increase could partly silence inflammation during metabolic stress and reinstate tissue integrity. For instance, ROR-γt SUMOylation suppresses transcription of IL-17 A, which amplifies inflammation and recruits neutrophils and monocytes via stimulating inflammatory cytokines production [144, 145].

SUMOylation participates in tumorigenesis via inflammatory pathways as well (Table 2). Recognized as an indispensable mediating factor in inflammatory pathways during carcinogenesis, NF-κB also acts as a TF, which is a nonnegligible target of SUMOylation. Simultaneous SUMOylation of Mesencephalic astrocyte-derived neurotrophic factor (MANF) and p65 by SUMO1 facilitate MANF nuclear translocation and their interaction, disrupting the NF-κB/Snail pathway and subsequent EMT in HCC [54]. TAK1 SUMOylation at K329 and K562 induced by TRIM60 suppresses MAPK/NF-κB activation via disturbing TRAF6/Table 2/TAK1 complex formation and the innate immune response [146]. In addition, IkappaBalpha (IκB) is modified by SUMO1 on K21, which acts as the site of ubiquitination as well. Therefore, SUMOylated IκB fails to enter signal-induced degradation, interfering with NF-κB signaling activation [147] . Comerford et al. also verified an analogous mechanism. Under normoxia, cAMP-response element-binding protein (CREB) and IκB are ubiquitinated, resulting in the induction of proinflammatory phenotype. While under hypoxia, modified by SUMO1, CREB, and IκB are stabilized, and inflammatory phenotype is subsequently inhibited [148]. However, the diametrical effect of NF-κB-related cascade reaction happens in that SUMO2/3-modifed IκBα is more susceptible to the proteasome [149]. Intriguingly, a regulatory loop emerges in NF-κB activity and its SUMOylation. That is PIAS3-mediated ReIA SUMOylation, a subunit of NF-κB, contributes to NF-κB inhibition, while its SUMOylation can be facilitated by NF-κB activation [150]. Furthermore, pro-apoptotic and anti-proliferative activities of interferon beta (IFNβ) were reported in BRCA. Decque et al. elucidated that SUMOylation silenced IFNβ and interferon alpha receptor 1 (IFNAR1) and thus restricted NF-κB associated cytokines production and Toll-like receptors (TLRs)-induced production of inflammatory cytokines. Therefore, SUMOylation of IFNβ tends to promote cancer cells’ malignant properties in diverse ways [151, 152, 153].

**Table 2:** Influences of SUMOylation in inflammatory TME

Inflammatory factors	SUMOylation-related participants	Mechanisms	Refs
NF-κBNF-κB/Snail	SUMO1	Inhibit NF-κBNF-κB/Snail and EMT in HCC through SUMOylating MANF and p65	[54]
MAPK/NF-κBNF-κB	TRIM60	Inhibit MAPK/NF-κBNF-κB and innate immune response via TAK1 SUMOylation	[146]
IκB/NF-κBNF-κB	SUMO1, UBC9	Inhibit NF-κBNF-κB activation via suppressing ubiquitination-induced IκB degradation or with the enhancement of RSUME	[147, 154]
CREB/IκB	SUMO1	Inhibit inflammatory processes via stabilizing CREB and IκB	[148]
IκB/NF-κBNF-κB	SUMO2/3, UBC9	Promote NF-κBNF-κB activity through facilitating IκB degradation	[149]
ReIA/NF-κBNF-κB	PIAS3	Induce NF-κB suppression through ReIA SUMOylation	[150]
IFNβ/IFNAR1, TLRs	SUMO1, SENP6	Restrain NF-κB and TLRs-associated cytokines production.	[151–153]
IL6	UBC9	Alleviate inflammatory infiltration via hub gene IL6	[155]
ROR-γt/IL17A	SUMO1, UBC9	Inhibit inflammation amplification and related cells recruitment via silencing IL-17 A transcription	[144, 145]
RelA, cFos, and cJun	UBC9	Inhibit inflammatory cytokines production through modifying Akt1	[156]
NEMO	SENP1	Inhibit TNF-α and IL-6 expression and NF-κB activation through eliminate NEMO SUMOylation	[103, 157]
NEMO	SENP2	Inhibit DNA damage induced NF-κB activation via NEMO deSUMOylation and form a negative feedback loop in response to genotoxic stimuli	[158
NEMO	SENP6	Inhibit NF-κB activation via Ikk deSUMOylation and CYLD mediated-degradation	[153]
CD45/STAT3/MDSC	SENP1	Inhibit MDSC expansion in several organs via CD45 deSUMOylation and STAT3 phosphorylation	[159]

UBC9 plays a significant role in the inflammatory infiltration of TME because of its uniqueness in the SUMO pathway. Given that RSUME increases IκB contents and stabilizes HIF-1α during cellular stress like heat shock and hypoxia, leading to inhibition of NF-κB. Researchers have found that this mechanism was implemented via RSUME-enhanced Ubc9 thioester formation, favoring noncovalent binding of SUMO1 to UBC9 and SUMO polymerization [92, 154]. In bladder cancer, Xia et al. determined different SUMOylation patterns and gene clusters that shape the TME and clinicopathological features [155]. UBC9 was found overexpressed in bladder cancer, and its downregulation contributed to obvious inflammatory gene activation. A prominent marker for cancer stem cells (CD44) is mediated by interleukin-6 (IL-6), which is a hub gene in UBC9 regulatory network. Therefore, UBC9 is to eliminate inflammatory infiltration, which might be a threat to tumorigenesis [155]. Likewise, compromised UBC9 function displays decreased AKT1 SUMOylation and increased proinflammatory cytokines including RelA, cFos, and cJun [156].

In addition to the E1, E2, E3 enzymes, and the SUMO molecular, SENP family, the deSUMOylation executors, mediate inflammatory processes as well. Yang et al. found that intermittent hypoxia (IH) could not only stimulate tumor necrosis factor-α (TNF-α) and IL-6 but also NEMO SUMOylation. While SENP1 attenuates NF-κB activation via disturbing NEMO SUMOylation [103, 157]. SENP2 also efficiently deSUMOylates NEMO, inhibiting DNA-damage-induced NF-κB activation and subsequently suppressing SENP1/2 transcription in response to genotoxic stimuli [158]. K277 of NEMO/Ikkγ is SUMOylated by SUMO2/3, which hinders the binding of CYLD/NEMO and therefore enhances the inhibitor of κB kinase (Ikk) activation. While deSUMOylation of NEMO by SENP6 can inhibit Ikk activation and subsequent IκB degradation, impairing NF-κB activation and proinflammatory genes expression [153]. Besides, CD45 SUMOylation via SENP1-deficiency facilitates STAT3 dephosphorylation, suppressing tumor development by myeloid-derived suppressor cells (MDSC) infiltration [159]. In the TME, various stimuli including chemotherapy or radiotherapy can trigger the release of pro-inflammatory mediators, turning “cold” tumors “hot”. Through SUMO modifications, tumor cells change the function of various pathways such as the NF-κB signaling pathway, which provides a chance for cancer treatment to target SUMOylation.

### SUMOylation participates in immune cells maturation and activation

Most tumor cells can be recognized by host CD8(+) T cells, and cancers that grow progressively must have escaped the antitumor attack. Recent research has illustrated two categories of tumor immune features in the TME, including innate immune activation which comprised immune cell infiltration and chemokine/IFN profile, and immune cell deficient phenotype. Tumor cells resist immune attack through the dominant immune-suppressive pathways and immune ignorance respectively [160]. Activation and inhibition of immune cells in the TME are critical for tumor cells to achieve immune escape, and molecules that regulate immune cells’ function usually undergo SUMOylation (Table 3).

**Table 3:** Functions of SUMO cascade in regulating activity of immune related cells

Immune cells regulators	SUMO-related participants	Mechanisms	Refs
PKM2	UBC9	Promote macrophage differentiation and TME remodeling through ectosomal excretion	[51]
IRF4	UBC9	Promote Treg cell proliferation and function upon TCR stimulation	[161]
BACH2	SENP3	Promote Treg cell stability maintenance via suppression of BACH2 SUMOylation and its nuclear export	[162]
NFATc1/IL-2	UBC9, PIAS1	Inhibit Treg expansion and promote autoimmunity through inhibiting IL-2 production	[163]
IL-7	UBC9	Promote T cells positive selection and late-stage maturation via maintaining CD8 single-positive cells survival	[165]
SLP-76	UBC9	Promote T cell activation via increasing IL-2 transcription	[166]
JunB/IL-2	UBC9	Promote T cell function via augmenting IL-2 and IL-4 expression	[167]
RORγt	SUMO3, PIAS4	Promote TH17 cells differentiation via maintaining its transcriptional activity by recruiting KAT2A and stabilizing the binding of SRC1	[168]
PLC-γ1	SUMO1, PIASxβ/3	Promote T cell activation through PLC-γ1 microclusters assembly	[169]
PKC-θ	PIAXxβ	Promote immune synapse formation and T cell activation	[170]
STAT5	SENP1	Promote early lymphoid precursors development via activating STAT5 signaling	[171]
Blimp-1	PIAS1	Promote plasma cells differentiation via enhancing interaction with HDAC2	[174]
DAXX	UBC9, SUMO1	Inhibit B cell development via nucleus localization and proper combination to PML oncogenic domains.	[172, 173]

Macrophage plasticity leads to either antitumor or protumor function in different conditions. SUMOylation of PKM2 induces monocyte-to-macrophage differentiation and TME remodeling by enhancing its exosomal excretion. Meanwhile, chemokines secreted by macrophages activate the CCL1-CCR8 axis, which promotes the PKM2-ARRDC1 combination and PKM2 excretion [51]. FOXP3-expressing regulatory T (Treg) cells eliminate aberrant immune response including anti-tumor immune response. Removal of Treg cells can restore anti-tumor immune function while Treg cell depletion may concurrently elicit autoimmunity. As a result, it is crucial to care about the exogenous adjustment of Treg cells among tumors. TCR stimulation enhances UBC9-mediated IRF4 SUMOylation, which augments Treg cell proliferation and regulates the downstream of TCR signals [161]. BTB domain and CNC homolog 2 (BACH2) functions as a mediator in primary adaptive immune response and immune deficiency. Yu and his colleagues suggested that SENP3 not only inhibited the nuclear export of BACH2 via deSUMOylation, maintaining Treg cell stability but also regulated ROS-induced immune tolerance [162]. NFATc1, a TF-regulating antigen receptor-mediated gene expression, is SUMOylated by UBC9 and PIAS1. Researchers constructed an NFATc1 SUMOylation deletion transgenic mouse and suggested that it exhibited increased IL-2 secretion, which promoted Treg expansion and impaired IL-17 and IFN-γ expression through STAT5 and Blimp-1 induction [163]. Intriguingly, Liu and his colleagues found that PIAS1 inhibited Treg cell differentiation independent of its SUMO E3 ligase activity. Mechanistically, PIAS1 binds to the Foxp3 promoter and increases histone H3 methylation, alleviating protein accessibility [164].

Apart from adoptive T cell therapy (ACT) whose function is instantaneous, endogenous T cells are more potent because of their ability to secure long-term memory with a broad repertoire of antigen specificity. SUMOylation was suggested to affect the development, activation, and function of T cells and B cells, thereby regulating the TME. Wang et al. selectively deleted UBC9 in T cells and found IL-7 signaling loss with increased apoptosis and attenuated proliferation during initial positive selection, resulting in defective late-stage maturation [165]. Immune adaptor SH2 domain containing leukocyte phosphoprotein of 76 kDa (SLP-76) is a substrate for SUMOylation cascade at K266/K284, and TCR stimulation facilitates SLP-76-UBC9 association, increasing the NFAT mediated IL-2 transcription [166]. JunB is another indispensable transcriptional activator of IL-2 and IL-4, regulating T cell function, and its SUMOylation on K237 also favors resting and activated primary T cells [167]. RORγt was found to mediate TH17 cell differentiation which regulated thymocyte development and lymph node genesis. While RORγt SUMOylation at K31 by SUMO3 and PIAS4 enhances its transcriptional activity, sustaining TH17 differentiation and CD8(+) T cell immature single-positive cells (ISPs) activity [168]. K54 of phospholipase C-γ1 (PLC-γ1) exhibits PIASxβ/PIAS3/SUMO1-regulated modification upon TCR stimulation, promoting PLC-γ1-microclusters assembly which favors T cell activation [169]. Likewise, kinase PKC-θ is SUMOylated via PIASxβ upon TCR, and its deSUMOylation impairs the coaccumulation of PKC-θ and CD28, inhibiting the formation of a mature immune synapse and T cell activation [170]. Moreover, SENP1-mediated SUMO2-STAT5 deSUMOylation facilitates its acetylation and downstream signaling, impelling the development of early T and B cells [171].

In plasma cells, SUMO1/UBC9-mediated Daxx SUMOylation was reported to not only impair the transcriptional potential of TFs but also facilitated type I IFN-regulated suppression of B cell development. Muromoto et al. constructed a SUMOylation-defective Daxx K630/631A mutant and found that the mutation transferred Daxx from the nucleus to cytoplasm and decreased the interaction with PML [172, 173]. Also, B lymphocyte-induced maturation protein-1 (Blimp-1) plays a crucial role during plasma cell differentiation that depends on PIAS1-induced Blimp-1 SUMOylation on K816, which increases the interaction with HDAC2 [174].

When present in the TME, SUMOylation influences tumor development via mediating immune cells’ activity, and this gives us a therapeutic hint that simultaneously targets SUMOylation and immune checkpoints. For example, irradiation (IR) + ATR inhibitors (ATRi) like berzosertib boosts the STING signaling and triggers strong innate immune activation by increasing SUMOylation at K127 of SHP1 [175]. Based on these, we suspect that whether SUMOylation could turn the “cold” TME “hot” to facilitate immune checkpoint inhibitor (ICI) therapy.

### SUMOylation participates in the exosomes-dependent dialog in the TME

The exosomes, extracellular vesicles secreted from all cells, largely contribute to the communication between the TME and cancer cells as well. Jena et al. illustrated that not only tumor cells secreted exosomes containing various cytokines, chemokines, and miRNAs that affected stromal cells’ maturation and differentiation but also stromal cell-derived exosomes had an influence on tumor cells [176]. Intriguingly, SUMOylation is also involved in intracellular communication through exosomes. SUMOylation of hnRNPA1 in BLCA facilitates the recognition of the lncRNA ELNAT1 via the endosomal sorting complex required for transport (ESCRT). ELNAT1 in the exosome is then transmitted into human lymphatic endothelial cells (HLECs), enhancing lymphangiogenesis by transcriptionally promoting SRY-box transcription factor 18 (SOX18) [64]. Analogously, SAE1/SUMO2-mediated hnRNPA1 SUMOylation in PDAC is elevated via KRASG12D mutation-induced hyperactivation of SUMOylation, and SUMOylated hnRNPA1 tends to be packaged into exosomes and transmitted to lymphatic endothelial cells, stabilizing prospero homeodomain protein 1 (PROX1) and promoting lymph node (LN) metastasis [177]. Furthermore, SUMOylation of hnRNPA1 in small extracellular vesicles (sEVs) was verified to steer sEV-miRNAs loading, which was suggested to promote communication between malignant cells and the TME, and thus enhance proliferation and metastasis [178]. It is noteworthy that the exact mechanism by which SUMOylation regulates extracellular vesicles is limited to hnRNPA1. Since a large number of proteins can be modified by SUMO, more SUMOylation-mediated communication by exosomes in the TME remains to be discovered.

### The future of SUMOylation

#### Orchestrating SUMOylation and the TME to resolve drug resistance

Nowadays, therapeutic resistance has become a thorny problem during cancer treatment. There is evidence that SUMOylation plays a pleiotropic role in the TME remodeling which dramatically affect drug resistance. In the hypoxic microenvironment of the EOC, SENP1 alleviates tumor cells’ sensitivity to chemotherapy via deSUMOylating HIF-1α and upregulating its expression [72]. The TME reprogramming in terms of metabolic reprogramming is also affected by SUMOylation, leading to chemotherapy resistance in PC. SENP1-mediated deSUMOylation of HK2 helps its mitochondria binding and consequently augments glucose consumption as well as lactate production [106]. In Irinotecan (CPT-11) resistant COAD cells, researchers also observed a dramatic increase of SUMO pathway members, accumulation of SENP1 and HIF-1α, and upregulation of glycolysis-relative protein markers, indicating the alterations of the metabolic microenvironment [179]. Demel and his colleagues found that SUMOylation activation inhibited MHC class I (MHC-I) antigen presentation, contributing to immune evasion from CD8(+) T cells and subsequent resistance to immunotherapies. Therefore, SUMOi is expected to restore antigen presentation machinery and augment the killing effect of CD8(+) T cells in the TME [180]. Moreover, a low SUMOylation level of SP1 contributes to SP1 and SNHG17 upregulation, which results in drug resistance through activating the Notch2 pathway in GC [181]. Although not mentioned by the authors, recent studies have shown the crucial role of the Notch2 pathway in TME remodeling in terms of regulating the anti-tumor infiltrate, which is likely to cause resistance [182]. Additionally, the SUMOylation-loss of PML triggers the abnormal NF-kB activation and is responsible for gemcitabine/oxaliplatin resistance in PDAC [183]. The evidence presented thus far supports that the SUMO pathway has the potential to modulate therapy resistance via TME remodeling, providing a window of opportunity for the application of the SUMOi to re-sensitize resistant individuals.

#### Applying SUMOi to clinical practice

**Fig. 4:**
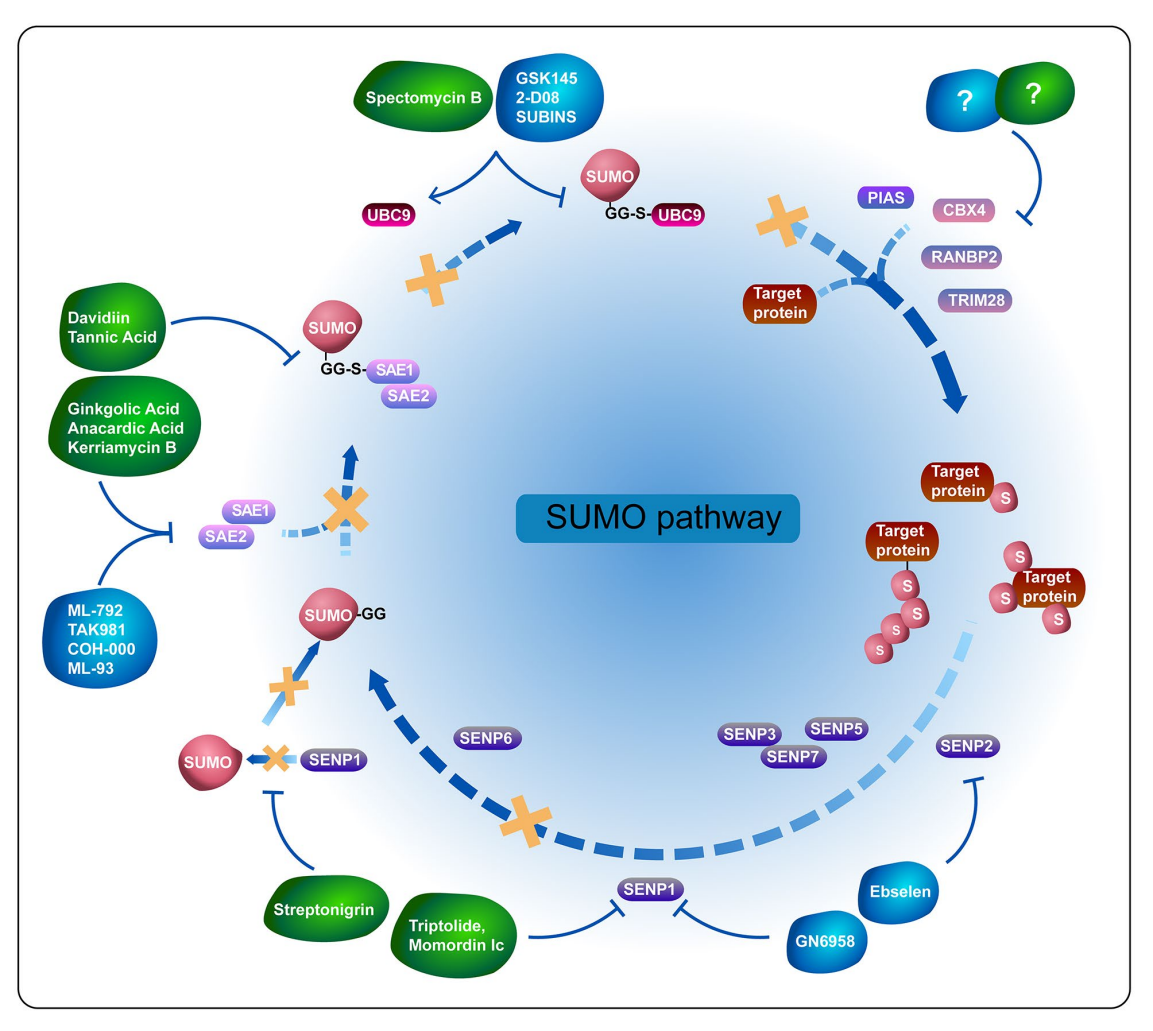
Functional sites of SUMOylation inhibitors. Ginkgolic acid, anacardic acid, and kerriamycin B blocks SAE1/2, while Davidiin and tannic acid impairs formation of the SAE1/2-SUMO intermediate. ML-792, TAK981, COH-000, and ML-93 inhibits SUMO E1 as well. Spectomycin B and GSK145, 2-D08, and SUBINS bind to UBC9, disturbing its interaction with SUMO. Triptolide, Momordin Ic and streptonigrin respectively inhibit SENP1 and disrupt SENP1-SUMO1 interaction. GN6958 and Ebselen suppress SENP1 and SENP2 respectively. There is no E3 inhibitor under research. Green inhibitors are natural compound while blue ones are synthetic product

The critical functions of SUMOylation in the TME provide opportunities for drug development and clinical trials. Recent research has displayed the potential of SUMOi for anticancer therapy. It has been confirmed that UBC9 or SAE1/2 depletion impaired cell survival through chromatin and non-chromatin-related manner, which has been identified through a proteomics mode as well [184, 185, 186]. As previously mentioned, SUMOylation cascade participants (E1, E2, E3-ligases, and SENPs) are upregulated in several tumors. Concurrently, they modify some proteins closely associated with the TME. These findings pave the way for the development of SUMOi that can be used to reverse the TME, thereby facilitating cancer treatment. Inhibitors targeting distinct parts of the SUMO circulation are shown in Fig. 4.

- E1 inhibitors.

Natural compounds blocking SAE1/2 were first reported in 2009, including ginkgolic acid, anacardic acid, and kerriamycin B [187, 188]. From 2014 to 2015, two other compounds appeared, that is, Davidiin and tannic acid. SUMO conjugations to target proteins are blocked by these compounds since they impair the formation of the SAE1/2-SUMO intermediate [189, 190]. A phenol in Ginkgo biloba L. named Ginkgolic acid was known to have antibacterial and antitumor properties through its targeting of proinflammatory factors such as prostaglandins and leukotrienes, suppressing the pro-tumor inflammatory microenvironment formation [191]. Given the poor specificity and side effects of these natural products, synthetic inhibitors of the SUMO E1 were reported one by one from 2017 to 2021, including ML-792, its derivative TAK981, COH-000, and ML-93. Their high specificity avoids additional effects on other PTMs, such as ubiquitylation and neddylation. It was reported that COH-000 bound to Cys30 of SAE2 in an allosteric site, and ML-792, TAK-981, and ML-93 disrupted SAE1/2 activity through forming an adduct with SUMO [59, 192, 193, 194]. There was also evidence that hyperactivation of MYC sensitized PDAC cells to ML-93-mediated SUMO inhibition. Moreover, ML-93 impedes metabolic reprogramming in the TME because MYC-driven metabolic signals on which malignancies depended are susceptible to ML-93 [137]. Crowl et al. found that SUMO2/3 perturbed IFN induction, which prevented the spread of inflammation [195]. Interestingly, the anti-lymphoma activity of TAK-981 relies on IFNAR as well. Furthermore, TAK-981 can weaken the immune escape ability of tumor cells by enhancing the presentation of exogenous antigens released by dying cancer cells and facilitating subsequent cytotoxic T cell initiation [196]. Identically, Kumar et al. confirmed that TAK-981 increased the proportions of activated CD8(+) T cells and natural killer (NK) cells through activating STAT1 and IFN target genes, strengthening immune surveillance of tumor cells [197]. Noteworthy, three clinical trials (NCT03648372, NCT04065555, and NCT04074330) concentrated on TAK-981 in patients respectively with metastatic solid tumors or lymphomas, head and neck cancer, and non-Hodgkin lymphoma. These trials extensively probed TAK-981 about its safety, tolerability, pharmacokinetics, efficiency in combination with cetuximab or avelumab, or rituximab, and its biological influences within the TME.

b. E2 inhibitors.

Natural product Spectomycin B and synthetic small molecular (GSK145A, 2-D08, and SUBINS) have emerged to inhibit SUMOylation through binding to and blocking UBC9-SUMO coalition [198, 199, 200, 201] 2′,3′,4′, -trihydroxyflavone (2-D08) that disrupts SUMO from transferring to substrates has been widely utilized in research. It was suggested that 2-D08 could induce apoptosis in AML cells and inhibit metastasis in PDAC via NOX2 and KRAS deSUMOylation respectively [202, 203]. Nevertheless, although various E3 ligases are liable to transfer SUMO to target proteins, there are no molecule inhibitors for E3 yet, which becomes a potential opportunity for exploration.

c. SENPs inhibitors.

Upregulation of SENP1 and SUMOylation disorder are favorable for PC progression. Natural product small molecules such as triptolide and Momordin Ic can optimize PC prognosis through inhibiting SENP1 [204, 205]. Streptonigrin, another natural product, can also disrupt SENP1-SUMO1 interaction, and thus alleviates HIF1α expression [206]. The synthesized product GN6958 and Ebselen keep SUMOylation to a certain stoichiometric level by suppressing the activity of SENP1 and SENP2 respectively [207, 208]. As a result, SENPs inhibitors are also expected to achieve better efficacy in vitro, which would pave the way for further preclinical studies.

### Challenges of future research on SUMOylation

**Fig. 5:**
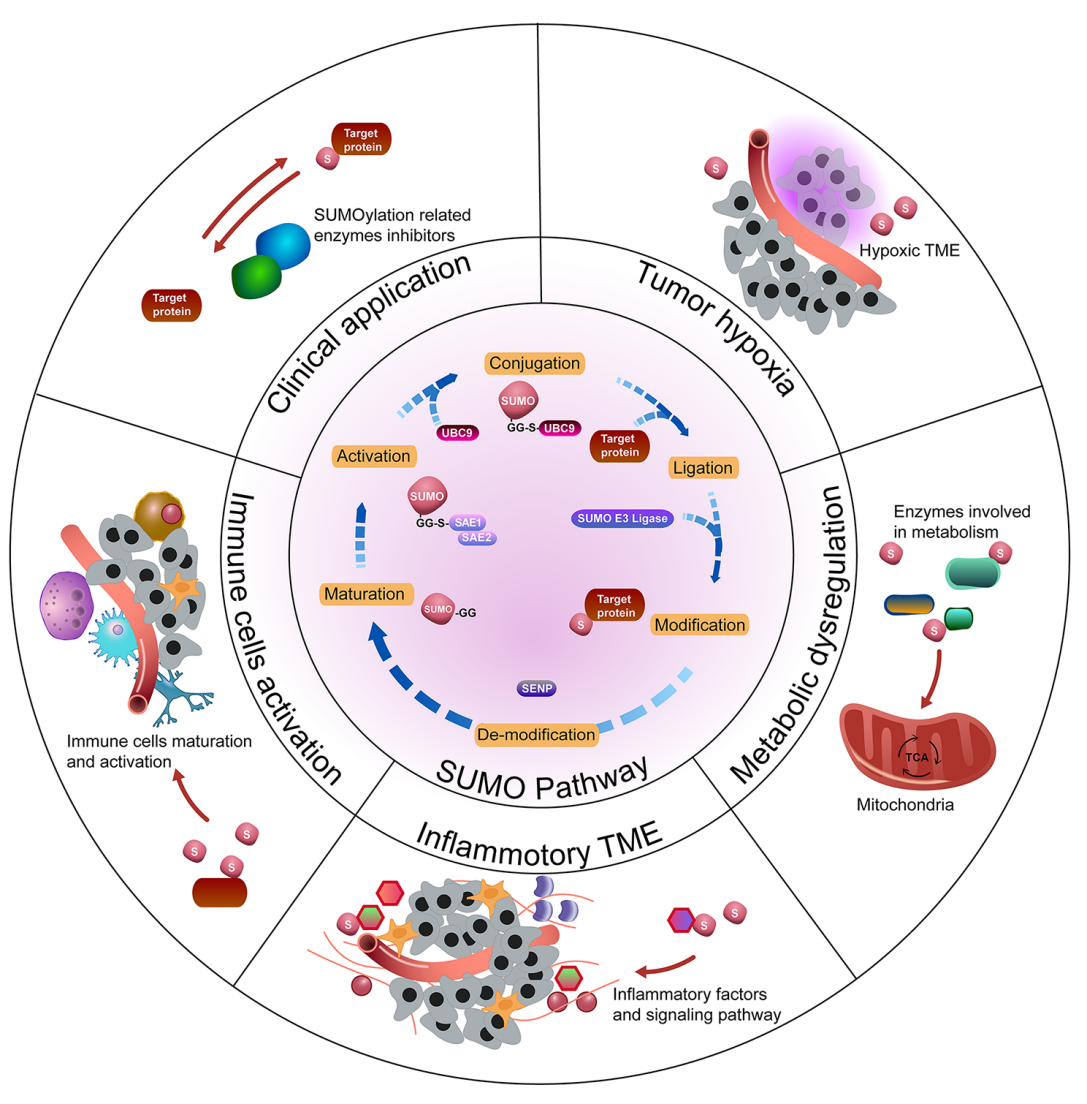
A summary of SUMOylation in the TME and therapeutic implications

Combined, studies concentrating on SUMOylation have unraveled the possible mechanism of its role in TME reprogramming and tumorigenesis, and discovered some potential inhibitors targeting SUMO circulation to hinder tumor development (Fig. 5). However, massive future work is required to reveal the functions of SUMOylation more comprehensively in the TME and expand its applications.

Firstly, to resolve the lack of natural protease sites in the C-terminal tail of SUMO proteins, a method using α-lytic protease, WaLP, was reported [209]. This method can generate peptides containing SUMO-remnant diglycyllysine (KGG) and detect endogenous SUMO modification at a proteome-wide and site level. However, this method fails to distinguish SUMO1-4, and it can’t differentiate between SUMOylated, Fatylated, or Fublylated proteins yet.

The second issue deserving attention is that the networks and pathways activated or inhibited by the “SUMO switch” lack more comprehensive interpretations. Additionally, whether global SUMO proteome alterations upon stress induce distinct biological processes via common signaling pathways hasn’t been clarified.

The third issue is that most potential medical intervention targeting SUMOylation is confined to TAK-981. Given that TAK-981 promotes the functions of T cells, we wonder if its combined effect with ICIs is more beneficial. Additionally, although family members of PIAS E3 enzymes can influence hypoxia and metabolic state in the TME and even activate immune cells as described above, there is no molecular inhibitor targeting E3 ligases yet. This defect might be attributed to the diversity of E3 enzymes. As a result, we expect an inhibitor that specifically targets E3 ligase-mediated processes. In short, much remains to be learned before we gain an insight into the reversible switch of the SUMO cascade and its role in the TME reprogramming.

## Conclusion

As one of the most prevalent modifications, SUMOylation is increasingly implicated in tumor initiation and progression. Recently, massive SUMOylated or deSUMOylated proteins were found to act as the executor of hypoxia adaption, metabolic reprogramming, inflammatory, and immune responses during the formation of the TME. Based on this, SUMOylation may serve as a promising diagnostic as well as therapeutic strategy benefiting from its availability of highly specific lyase and high-throughput proteomics.

## Data Availability

Not applicable.

## List of abbreviations

ACSS: Acetyl-coenzyme A synthetase
AMPK: 5’-AMP-activated protein kinase
ARNT: Aryl hydrocarbon receptor nuclear transporter
ARRDC1: Arrestin domain containing 1
BACH2: BTB domain and CNC homolog 2
BHLHE40: Basic helix-loop-helix family member e40
BLCA: Bladder cancer
Blimp-1: B lymphocyte-induced maturation protein-1
BRCA: Breast cancer
CBP: CREB binding protein
CBX4: chromobox 4
CCA: Cervical cancer
CCL1: C-C motif chemokine ligand 1
CCR8: C-C motif chemokine receptor 8
CLDN6: Claudin 6
COAD: Colon cancer
CREB: cAMP-response element-binding protein
CRMP2: Cysteine repeat modular protein 2
CSN5: COP9 signalosome subunit 5
Daxx: Death domain-associated protein
DDR: DNA damage response
DSB: DNA double-strand breaks
ECV: Elongins and Cullins
EMT: Epithelial-mesenchymal transition
EOC: Epithelial Ovarian Cancer
ESCC: Esophageal squamous cell carcinoma
ESCRT: Endosomal sorting complex required for transport
FASN: Fatty acid synthetase
FIH: Factor inhibiting HIF
FOXM1b: Forkhead box M1b
FOXO: Forkhead box O
FOXP3: Forkhead Box Protein P3
FTO: Fat mass- and obesity-associated gene
GATA1: GATA binding protein 1
GBM: Glioblastoma
GC: Gastric cancer
GLUT1: Glucose transporter 1
GNAO1: Guanine nucleotide-binding protein G (o) subunit alpha
GSCs: Glioma stem cells
GSH: Glutathione
HAF: Hypoxia-associated factor
HDAC6: Histone deacetylase 6
HIF-1: Hypoxia inducible factor 1
HK2: Hexokinase 2
HLECs: Human lymphatic endothelial cells
HnRNPK: Heterogeneous nuclear ribonucleoprotein K
hnRNPD: Heterogeneous nuclear ribonucleoprotein D
HPV: Human Papilloma Virus
HRE: Hypoxia responsive elements
HSP27: Heat shock protein 27
HTERT: Human telomerase reverse transcriptase
ICI: Immune checkpoint inhibitor
IFNβ: Interferon beta
IGF-1R: Insulin-like growth factor 1 receptor
IκB: IkappaBalpha
IL-2: Interleukin 2
IRF-1: Interferon regulatory factor 1
ISPs: Immature single-positive cells
LDH1: Lactate dehydrogenase 1
LN: Lymph node
m5C: 5-methylcytosine
m^6^A: N6-Methyladenosine
MANF: Mesencephalic astrocyte-derived neurotrophic factor
MANF: Mesencephalic astrocyte-derived neurotrophic factor
MCM: Minichromosome maintenance
MDM2: Murine double minute 2 homologue
MDSC: Myeloid-derived suppressor cells
METTL3: Methyltransferase 3
mTORC1: Rapamycin (mTOR) complex 1
NADPH: Nicotinamide adenine dinucleotide phosphate
NDRG2: N-myc downstream regulated gene 2
NEMO: Nuclear factor (NF)-kappaB essential modulator
NFATC1: Nuclear factor of activated T cells 1
NF-κBNF-κB: Nuclear Factor Kappa-Β
NPC: Nasopharyngeal carcinoma
NRF2: Nuclear factor erythroid-2 related factor 2
NSUN2: NOP2/Sun RNA methyltransferase 2
OSBPL3: Oxysterol binding protein-like 3
OSCC: Oral squamous cell carcinoma
PDK1: Pyruvate dehydrogenase kinase 1
PDPK1: Phosphoinositide-dependent protein kinase 1
PGK1: Phosphoglycerate kinase 1
PHDs: Prolyl hydroxylase domain containing proteins
PI3K: Phosphoinositide 3-kinase
PIAS: Protein inhibitor of activated STAT
PKC: Protein kinase C
PKM2: Pyruvate kinase isoenzyme M2
PLC-γ1: Phospholipase C-γ1
PML: Promyelocytic leukemia
PPARα: Peroxisome proliferator-activated receptor alpha
PROX1: Prospero homeodomain protein 1 (PROX1)
PC: Prostate cancer
PSTAR: P53-stabilizing and activating RNA
PTEN: Phosphatase and tension homolog
PTM: Post-translational modification
RanBP2: RAN binding protein 2
RCC: Renal cell cancer
RGS12: Regulator of G protein signaling 12
RNF4: Ring finger protein 4
ROS: Reactive oxygen species
RSUME: RWD domain-containing protein SUMO Enhancer
SCLC: Small cell lung cancer
SENPs: Sentrin-specific Proteases
sEV: Small extracellular vesicle
SIMs: SUMO-interacting motifs
SIRT1: Sirtuin 1
SNAI2: Snail family zinc finger 2
SnoN: SKI like proto-oncogene
SOX18: SRY-box transcription factor 18
SP1: Specificity protein 1
SREBP: Sterol regulatory element-binding proteins
STAT: Signal transducer and activator of transcription
SUMO: Small ubiquitin-related modifier
TCA: Tricarboxylic acid
TLRs: Toll-like receptors
TME: Tumor microenvironment
TNBC: Triple-negative breast cancer
TRIM28: Tripartite motif containing 28
VEGFR2: Vascular endothelial growth factor receptor 2
VHL: Von Hippel-Lindau
YTHDF2: YTH N6-methyladenosine RNA binding protein 2
ZEB1: Zinc finger E-box binding homeobox 1
